# The preliminary study on cardiac structure and function in Chinese patients with primary hyperparathyroidism

**DOI:** 10.3389/fendo.2023.1083521

**Published:** 2023-02-07

**Authors:** Rong Chen, An Song, Ou Wang, Yan Jiang, Mei Li, Weibo Xia, Xue Lin, Xiaoping Xing

**Affiliations:** ^1^ Department of Endocrinology, Key Laboratory of Endocrinology, National Health Commission, Peking Union Medical College Hospital, Chinese Academy of Medical Science & Peking Union Medical College, Beijing, China; ^2^ Department of Cardiology, Peking Union Medical College Hospital, Chinese Academy of Medical Science & Peking Union Medical College, Beijing, China

**Keywords:** primary hyperparathyroidism, echocardiography, cardiac structure, cardiac function, China

## Abstract

**Purpose:**

Recent evidences show that primary hyperparathyroidism (PHPT) patients have a high prevalence of cardiovascular diseases. However, the reported changes in cardiac status are inconsistent in previous studies. The present work evaluated the cardiac structure and function in PHPT patients by echocardiography.

**Methods:**

PHPT patients and age- and sex-matched healthy controls were enrolled in this case-control study. Biochemical parameters were retrospectively collected from PHPT patients. Cardiac function and structure were assessed in all subjects using echocardiography.

**Results:**

A total of 153 PHPT patients and 51 age- and sex-matched healthy controls were enrolled in this study. The mean serum calcium and parathyroid hormone (PTH) levels in PHPT patients were 2.84 ± 0.28mmol/L and 206.9 (130.0, 447.5) pg/ml, respectively. Left ventricular ejection fraction (LVEF) and early to late mitral annular velocity (E/A) were significantly lower in PHPT patients than in healthy controls (68.2 ± 6.0 vs. 70.7 ± 16.7%, 1.0 ± 0.5 vs. 1.4 ± 0.5, respectively, *p* both < 0.05). The left ventricular mass index (LVMI) and the relative wall thickness (RWT) were not significantly different between the two groups. However, the difference in LVEF between PHPT patients without hypertension and diabetes and the control groups disappeared. The majority of PHPT patients had normal cardiac geometry; however, a proportion of them exhibited concentric remodeling (normal LVMI, RWT≥0.42). Serum calcium, corrected calcium, ionized calcium and PTH were inversely related to E/A, whereas serum phosphorus and 24-hour urine calcium were positively related to E/A. Furthermore, biochemical parameters were not correlated with LVEF.

**Conclusions:**

These findings demonstrate that PHPT patients exhibit diastolic cardiac dysfunction reflected by decreased E/A, as well as possible cardiac structural abnormalities. The serum calcium, phosphorus, and parathyroid hormone levels may influence cardiac structure and function.

## Introduction

Primary hyperparathyroidism (PHPT) is the third most prevalent endocrine disorder in Western countries after diabetes mellitus and thyroid diseases, characterized by increased calcium levels combined with elevated or inappropriately normal parathyroid hormone (PTH) levels (1, 2). Asymptomatic PHPT has become increasingly widespread in China as general health checkups and routine biochemical screening have become more popular. Previous investigations indicate an increase proportion of asymptomatic PHPT patients in Shanghai, China, from 5.9% to 35.0% from 2005–2007 to 2017–2019 (3, 4). Increased serum calcium and PTH concentrations affect multiple organs, manifesting as bone resorption or osteoporosis, kidney stone, renal function impairment, peptic ulcer, and so on (5, 6). Recent evidences show that non-classical manifestations of PHPT, including the involvement of cardiovascular system, have attracted increased attentions. However, according to the latest PHPT recommendations from the 4^th^ international workshop (7), it is stated that the existence and reversibility of PHPT cardiovascular symptoms remain unresolved concerns.

Studies have revealed that PHPT patients having cardiac structural and functional abnormalities are characterized by a higher left ventricular mass index (LVMI) and a lower ratio of early diastolic mitral inflow velocity to late diastolic mitral inflow velocity (E/A) than controls (8–10). LVMI has been extensively investigated as a risk factor for cardiovascular mortality in the general population, with a few studies reporting increased LVMI in PHPT (9, 11). The E/A is a common parameter used to assess diastolic ventricular function. Furthermore, a few researchers have discovered that parathyroidectomy potentially improve cardiac structure and function (12, 13). However, there are conflicting findings on the abnormality of cardiac structure and function in PHPT patients. A prospective case-control study has found that PHPT patients without cardiovascular risk factors show no difference in cardiac morphology and function, compared to the age-matched healthy controls (14). Elsewhere, an investigation looking into the cardiovascular effects of PTH has revealed that LVMI and E/A ratios are not significantly different between healthy and PHPT subjects (15). In addition, several studies have found no significant difference in the echocardiographic parameters before and after parathyroidectomy (9, 15, 16).

Most of the existing studies have small sample sizes (n=20-100), and the echocardiogram changes in PHPT are still controversy. Furthermore, the clinical profiles of PHPT patients have been demonstrated to vary by region. Liu et al. and Meng et al. have found that Chinese PHPT patients have more severe PHPT clinical phenotypes than Americans (3, 17). Research on cardiovascular manifestations in Chinese PHPT patients is largely scarce. The present investigation primarily aimed to evaluate cardiac structure and function using echocardiography in PHPT patients and analyze the factors influencing cardiac structure and function.

## Materials and methods

### Subjects

A total of 272 PHPT patients admitted to the endocrine ward in Peking Union Medical College Hospital (PUMCH) between January 2015 and December 2021 were enrolled in the study. PHPT was diagnosed by hypercalcemia combined with increased or inappropriately normal intact PTH level. Asymptomatic PHPT was defined as PHPT lacking apparent signs or symptoms of hypercalcemia or high levels of parathyroid hormone (18), such as gastrointestinal disorders, osteoporosis, fragility fractures, nephrolithiasis and nephrocalcinosis. Inclusion criteria were as follows: patients aged 18 and over; patients having received preoperative cardiac ultrasound examination; patients with complete clinical data. Familial or syndromic hyperparathyroidism, such as multiple endocrine neoplasia, familial hypocalciuric hypercalcemia and hyperparathyroidism-jaw tumor syndrome, were excluded based on medical history in combination with laboratory and imaging examination. Patients with coronary artery disease, cardiomyopathy and chronic systemic diseases, such as severe hepatorenal disease, were also excluded. Finally, 153 PHPT patients were included in this investigation, whereas 51 age- and sex-matched healthy subjects with echocardiograms were recruited from the PUMCH health examination center as the control group. All the healthy controls had no history of systemic diseases, including metabolic bone diseases. Serum biochemical indicators such as liver and kidney function, glucose, calcium, and PTH levels were all within normal ranges. This study was approved by the Ethics Committee of PUMCH and conducted in accordance with the principles in the Declaration of Helsinki.

### Clinical parameters

Data on demographics and medical history were obtained from PUMCH medical records, including gender, age, duration of PHPT, and target organ involvement of PHPT such as nephrolithiasis, nephrocalcinosis, subperiosteal resorption, osteoporosis and fragility fractures, etc. Lateral cephalogram and anteroposterior hand X-rays were used to assess the existence of subperiosteal bone resorption in PHPT patients. Bone mineral density (BMD) was measured by Dual-energy X-ray absorptiometry (American GE-Lunar). The osteoporosis diagnosis was based on BMD. A T score of -2.5 or less indicated osteoporosis in postmenopausal women or men 50 years and older. Z scores ≤ - 2.0 were classified as “below the expected range for age” for premenopausal women or men less than 50 years old. Fractures were assessed by the clinical history and lateral spine X-ray. Urolithiasis or renal calcification was evaluated using ultrasound. Anthropometric indexes were measured by the trained physicians, including height, weight, and heart rate. Body mass index (BMI) was calculated by dividing weight in kilograms by height in square meters. Body surface area (BSA) was calculated using Du Bois’ formula (19).

### Laboratory parameters

PHPT patients had their fasting venous blood samples collected in the morning. Total serum calcium, serum phosphorus, albumin, serum alkaline phosphatase (ALP), serum creatinine, and total 25-hydroxyvitamin (25OHD) were measured using an automated biochemical analyzer (Beckman Coulter AU5800, USA). Albumin-adjusted total calcium was calculated using the albumin correction formula (20): [40-albumin(g/l)]*0.02+total serum calcium (mmol/L). Plasma-ionized calcium was quantified using a radiometer ABL800 FLEX blood-gas analyzer (ABL800 FLEX, Denmark). Serum β-C-terminal peptide of type I collagen (β-CTX) and intact PTH were quantified using chemiluminescence immunoassay (Siemens ADVIA Centaur, Germany). The 24-hour urine calcium was tested using the NM-BAPTA assay (Roche Cobas c702, Switzerland). The intra-assay and inter-assay coefficients of variations (CVs) in 25OHD were 5.9% and 6.5%, respectively. PTH had intra-assay and inter-assay CVs of 2.6% and 5.8%, respectively. The intra-assay and inter-assay CVs for the other laboratory parameters were < 3.5%.

### Transthoracic echocardiography

All subjects underwent echocardiographic examinations; they were evaluated by a group of senior cardiologists using a transthoracic echocardiogram (VIVID E9, GE Ultrasound, USA) with an m5s-D probe. Echocardiographic measurements were taken in Doppler mode and M-mode (21). Parameters, including interventricular septum end-diastolic thickness (IVSd), left ventricular posterior wall end-diastolic thickness (LVPWd), left ventricular end-diastolic diameter (LVEDD), left ventricular end-systolic diameter (LVESD), left ventricular fractional shortening (FS) and left ventricular ejection fraction (LVEF), were all measured. Left ventricular mass (LVM, grams) was calculated using the method described by the American Society of Echocardiography (22). LVM index (LVMI, g/m^2^) was calculated by dividing LVM by BSA. Abnormal LVMI is defined as a value greater than 95 g/m^2^ in women and 115 g/m^2^ in men. The relative wall thickness (RWT) was calculated as follows: (2 × LVPWd)/LVEDD. The combination of RWT and LVMI classified cardiac morphology into four types: normal geometry, concentric remodeling, eccentric hypertrophy, and concentric hypertrophy (21). RWT permitted categorization of normal LVMI as normal geometry (RWT ≤ 0.42) or concentric remodeling (RWT>0.42), and an increase in LVMI to be classified as concentric (RWT>0.42) or eccentric (RWT ≤ 0.42) hypertrophy. The E/A was calculated using the Doppler mode.

### Statistical analysis

Continuous variables were presented as mean ± standard deviation or median and inter-quartile ranges if not normally distributed. Continuous variables between the PHPT and control groups were compared using the student’s independent t-test or Mann-Whitney test. Categorical variables were expressed as numbers or percentages. The correlations between laboratory parameters and echocardiographic variables in PHPT group were examined using Hierarchical linear regression analysis, being adjusted for age, gender, duration of PHPT, BMI, hypertension, and diabetes mellitus. All data were analyzed using SPSS software version 19.0 (Chicago, IL, USA). *p* < 0.05 denoted statistical significance.

## Results

### General clinical characteristics of healthy controls and PHPT patients

A total of 153 PHPT subjects were included in the study (**Table 1**). The PHPT and control groups had similar gender distributions (women 52.9%, men 47.1%). The mean ages of the PHPT and control groups were 56.4 ± 10.5 years and 57.0 ± 11.0 years, respectively. The PHPT group had 55 (35.9%) patients with hypertension and 31(20.3%) with diabetes mellitus. Nephrolithiasis and nephrocalcinosis presented in 56 and 10 PHPT patients, respectively. Radiological evidence of subperiosteal resorption was revealed in 21 PHPT patients. Osteoporosis and fractures were reported in 70 and 24 PHPT patients, respectively. Moreover, nearly 26% of PHPT patients were asymptomatic. The median duration of PHPT was 2.0 (0.5, 6.0) years. The serum calcium and plasma ionized calcium were 2.84 ± 0.28 and 1.43 ± 0.14mmol/L, respectively, and serum PTH was 206.9 (130.0, 447.5) pg/ml in PHPT group. PHPT patients had significantly higher weight, BMI, and heart rate than controls.

**Table 1:** Demographical, physical examination, and echocardiographic parameters in PHPT and healthy control subjects.

	PHPT (n=153)	Control (n=51)	*p*
Gender (F/M)	81/72	27/24	1.000
Age (y)	56.4 ± 10.5	57.0 ± 11.0	0.712
Duration of PHPT (y)	2.0(0.5, 6.0)	–	–
Height (cm)	164.1 ± 8.8	164.5 ± 7.1	0.770
Weight (kg)	66.3 ± 12.5^*^	62.4 ± 8.8	0.039
BMI (kg/m^2^)	24.5 ± 3.2^*^	23.0 ± 2.1	0.000
Heart rate (per min)	79.1 ± 11.4^*^	68.4 ± 10.2	0.000
Hypertension (n/%)	55/35.9%	0	–
Diabetes mellitus (n/%)	31/20.3%	0	–
Serum calcium (mmol/L)	2.84 ± 0.28	–	–
Corrected calcium (mmol/L)	2.76 ± 0.31	–	–
Plasma ionized calcium (mmol/L)	1.44 ± 0.14	–	–
Serum albumin (g/L)	44.0 ± 5.0	–	–
Serum phosphorus (mmol/L)	0.80 ± 0.18	–	–
Serum PTH (pg/ml)	206.9(130.0, 447.5)	–	–
Serum ALP (U/L)	174.2 ± 230.1		
Serum β-CTX (ng/ml)	1.18 ± 0.90		
Serum 25OHD (ng/ml)	14.7 ± 6.7	–	–
Serum creatinine (umol/L)	76.5 ± 28.1	–	
24hUCa(mmol)	8.2 ± 4.0	–	–
LVEDD(mm)	46.4 ± 4.0	46.8 ± 4.1	0.565
FS(%)	38.4 ± 4.5^*^	40.4 ± 5.6	0.020
LVEF(%)	68.2 ± 6.0^*^	70.7 ± 6.7	0.012
LVMI(g/m^2^)	73.5 ± 17.7	74.5 ± 12.8	0.726
RWT(mm)	0.35± 0.05	0.36 ± 0.07	0.595
LVSd(mm)	8.3 ± 1.4	8.0 ± 1.1	0.104
LVPWd(mm)	8.2 ± 1.1	8.3 ± 1.3	0.456
E/A	1.0 ± 0.5^*^	1.4 ± 0.5	0.000

The date represents the means ± SD or median(interquartile).

*p < 0.05 was considered a statistically significant group difference between PHPT and control. The normal range of E/A was >0.8 in both females and males.

PHPT Primary hyperparathyroidism, BMI body mass index, PTH parathyroid hormone, 25OHD serum 25-hydroxyvitamin, ALP alkaline phosphatase, β-CTX β-C-terminal peptide of type I collagen, 24hUCa 24-hour urine calcium, LVEDD left ventricular end-diastolic diameter, FS left ventricular fractional shortening, LVEF left ventricular ejection fraction, LVMI left ventricular mass index, RWT relative wall thickness, IVSd interventricular septum end-diastolic thickness, LVPWd left ventricular posterior wall end-diastolic thickness, E/A ratio of early diastolic mitral inflow velocity to late diastolic mitral inflow velocity.

### Echocardiographic parameters and cardiac geometry in PHPT patients and controls

As illustrated in **Table 1**, the PHPT group reported significantly lower FS than the control group (38.4 ± 4.5% vs. 40.4 ± 5.6%, *p*=0.020). The LVEF was significantly lower in the PHPT group than in the control group (68.2 ± 6.0% vs. 70.7 ± 6.7%, *p*=0.012). The PHPT group had a significantly lower E/A compared with the controls (1.0 ± 0.5 vs. 1.4 ± 0.5), with a *p*-value less than 0.001. LVMI and RWT were not significantly different between the PHPT patients and controls. The PHPT group included 83 patients without hypertension and diabetes mellitus, who had significantly lower LVMI, LVPWd, and E/A than the healthy controls (LVMI 69.4 ± 15.2 vs. 74.57 ± 12.8g/m^2^, *p*=0.040; LVPWd 8.0 ± 1.1 vs. 8.3 ± 1.3mm, *p*=0.012; E/A 1.0 ± 0.3 vs. 1.3 ± 0.4, *p*<0.001). The LVEF showed no disparity between this subset of PHPT patients and controls. Furthermore, symptomatic and asymptomatic PHPT patients showed no statistical difference in terms of LVMI, RWT, LVEF, and E/A.

Of the 122 PHPT patients without diabetes, 39 had hypertension. Hypertensive PHPT patients showed significantly higher LVMI and RWT (LVMI 81.5 ± 18.9 vs. 69.4 ± 15.2g/m^2^, *p*<0.001; RWT 0.37 ± 0.05 vs. 0.35 ± 0.05, *p*=0.002), as well as significantly lower E/A (E/A 0.81 ± 0.24 vs. 1.02 ± 0.33, *p*<0.001) compared with the patients without hypertension. There was no difference in LVEF between hypertensive and normotensive PHPT patients (LEVF 68.2 ± 6.6 vs. 68.4 ± 5.2%, *p*=0.816).

**Figure 1:**
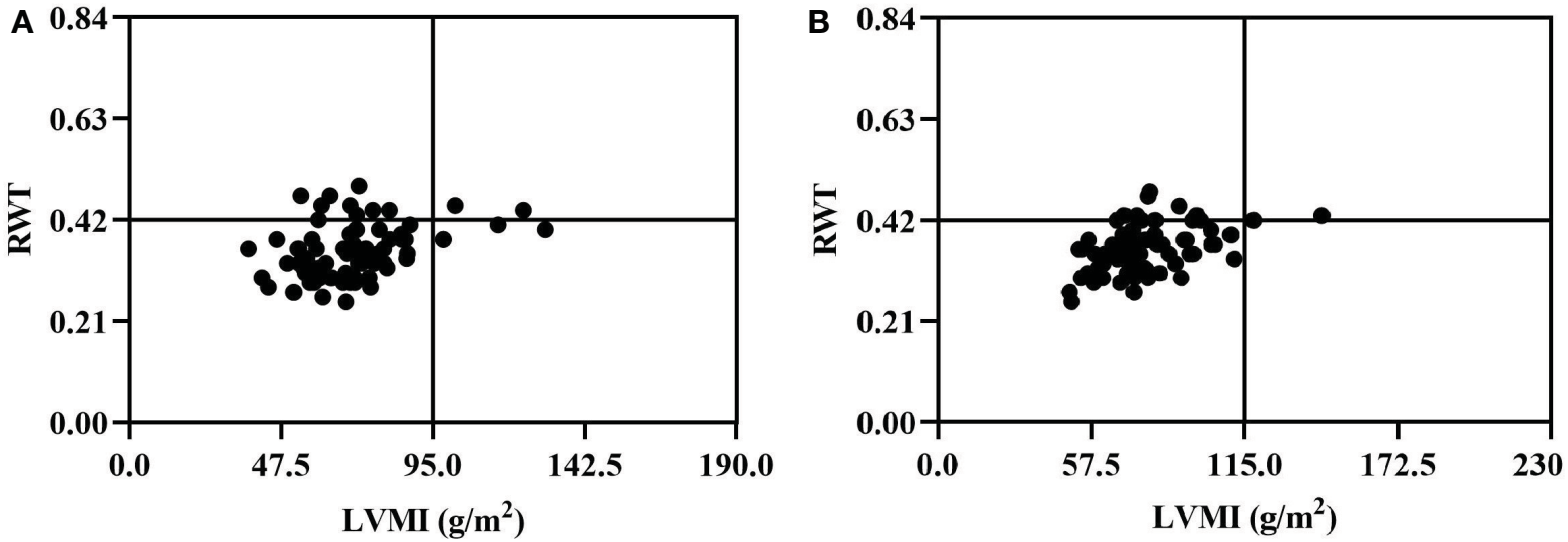
Cardiac geometry of female **(A)** and male **(B)** patients in 153 PHPT. The normal range of RWT was less than 0.42 in both females and males, and the normal range of LVMI was less than 95.0 g/m^2^ in women and less than 115.0 g/m^2^ in men. PHPT, Primary hyperparathyroidism; LVMI, left ventricular mass index; RWT, relative wall thickness.

**Figure 1** depicts the main characteristics of the cardiac geometry of female and male PHPT patients. A majority of PHPT patients had RWT less than 0.42 and normal LVMI. Among the 81 females, 75 had normal LVMI (less than 95 g/m^2^), and 66 had RWT less than 0.42. Six female patients had LVMI greater than 95 g/m^2^, two of whom had RWT greater than 0.42. Among the 72 male patients, 69 had normal LVMI (less than 115 g/m^2^), and 58 had RWT less than 0.42. Three male patients had LVMI greater than 115 g/m^2^ and RWT greater than 0.42.

### Determinants of the echocardiographic parameters in the PHPT group

**Table 2** shows the correlations between biochemical and echocardiographic parameters in the PHPT cohort. Notably, after adjusting for age, gender, BMI, duration of PHPT, hypertension, and diabetes, the corrected calcium and PTH levels were positively correlated with LVMI (r =0.164, *p*=0.036, r=0.298, *p*<0.001, respectively), whereas serum phosphorus was negatively correlated with LVMI (r=-0.167, *p* =0.044). Serum phosphorus was also negatively related to RWT (r=-0.230, *p*=0.008). There was no correlation established between biochemical parameters with LVEF. In addition, serum calcium, corrected calcium, plasma ionized calcium, and PTH were negatively correlated with E/A (r =-0.215, r =-0.243, r = -0.242, r = -0.234, all *p* were<0.05). Serum phosphorus and 24-hour urine calcium were positively correlated with E/A (r =0.188, r =0.169, *p*<0.05).

**Table 2:** The hierarchical linear regression analysis between biochemical and echocardiographic parameters in 153 PHPT patients after adjustments of confounding factors.

Echocardiographic parameters	Biochemical parameter	R^2^	ΔR^2^	ΔF	Standardization coefficient	*p*
Model	–	0.125	0.125	3.463	–	0.003
LVMI	Ca	0.142	0.017	2.904	0.134	0.090
	cCa	0.151	0.026	4.467	0.164	0.036
	iCa	0.132	0.008	1.278	0.091	0.260
	Pi	0.149	0.024	4.126	-0.167	0.044
	PTH	0.207	0.082	15.023	0.298	0.000
	ALP	0.154	0.029	5.040	0.179	0.056
	β-CTX	0.136	0.011	1.891	0.109	0.171
	25OHD	0.126	0.001	0.176	-0.035	0.676
	24hUCa	0.128	0.003	0.549	0.060	0.460
Model	–	0.042	0.042	1.062	–	0.388
RWT	Ca	0.049	0.007	1.042	0.084	0.309
	cCa	0.057	0.016	2.405	0.127	0.123
	iCa	0.043	0.001	0.136	0.031	0.713
	Pi	0.088	0.046	7.308	-0.230	0.008
	PTH	0.049	0.007	1.117	0.089	0.292
	ALP	0.043	0.001	0.184	0.036	0.668
	β-CTX	0.042	0.001	0.077	-0.023	0.782
	25OHD	0.058	0.016	2.514	-0.137	0.115
	24hUCa	0.042	0.000	0.001	-0.002	0.977
Model	–	0.049	0.049	1.255	–	0.282
LVEF	Ca	0.049	0.000	0.012	-0.009	0.913
	cCa	0.049	0.000	0.007	-0.007	0.933
	iCa	0.050	0.000	0.071	-0.023	0.790
	Pi	0.052	0.003	0.525	0.063	0.470
	PTH	0.050	0.001	0.125	0.030	0.724
	ALP	0.055	0.006	0.948	0.082	0.332
	β-CTX	0.054	0.005	0.796	0.074	0.374
	25OHD	0.054	0.005	0.762	0.076	0.384
	24hUCa	0.049	0.000	0.018	-0.012	0.892
Model	–	0.228	0.228	7.182	–	0.000
E/A	Ca	0.272	0.044	8.824	-0.215	0.003
	cCa	0.285	0.057	11.584	-0.243	0.001
	iCa	0.282	0.054	10.895	-0.242	0.001
	Pi	0.258	0.031	5.979	0.188	0.016
	PTH	0.278	0.051	10.171	-0.234	0.002
	ALP	0.234	0.006	1.127	-0.081	0.290
	β-CTX	0.241	0.013	2.518	-0.118	0.115
	25OHD	0.247	0.019	3.584	0.146	0.060
	24hUCa	0.254	0.026	5.045	0.169	0.026

ΔR^2^ R Square Change, ΔF F Change, Standardization coefficient represents the change of the dependent variable every time the independent variable changes a unit.

Model, adjusted for age, gender, duration of PHPT, body mass index, hypertension, and diabetes mellitus.

PHPT Primary hyperparathyroidism, LVMI left ventricular mass index, RWT relative wall thickness, LVEF left ventricular ejection fraction, E/A ratio of early diastolic mitral inflow velocity to late diastolic mitral inflow velocity, Ca serum calcium, cCa corrected calcium, iCa serum ionized calcium, Pi serum phosphorus, PTH parathyroid hormone, ALP alkaline phosphatase, β-CTX β-C-terminal peptide of type I collagen, 25OHD serum 25-hydroxyvitamin, 24hUCa 24-hour urine calcium.

## Discussion

PHPT in China is not as frequent as in Western countries, and their clinical spectrums differ significantly (3, 23). This is the first and relatively large sample-size investigation in mainland China to examine changes in cardiac structure and function in PHPT patients, which could provide evidence for future research and treatment decisions. This study suggested that PHPT patients had lower E/A than controls, and their serum calcium and PTH levels were inversely correlated with E/A. Moreover, PHPT patients showed a tendency towards concentric remodeling.

In this case-control research, we found no difference in LVMI between PHPT and healthy control groups, which corroborated with previous studies. In comparative research (14), Farahnak et al. also did not find a significant increase in LVMI in mild PHPT patients without cardiovascular risk factors/diseases compared to age-matched healthy controls. Jessica and colleagues (24) discovered no significant differences in LVMI between normo-calcemic PHPT, PHPT, and control groups in subjects without high cardiovascular risk. On the other hand, previous research findings into the change of LVMI in PHPT remained inconsistent. Kepez (9) found that LVMI was higher in PHPT patients (n=22) than controls in a small-scale investigation examining left ventricular performance in patients with PHPT. A recent study by Purra (8) found that symptomatic PHPT patients (n=100) had significantly higher LVMI compared with healthy controls when the two groups were matched for cardiovascular system risk factors. The differences can be explained by differences in subject recruiting conditions. The variations in LVMI in PHPT patients, as well as the effects of PTH on the cardiovascular system, warrant further investigation. In the present work, LVMI was found to be higher in PHPT patients with hypertension than in PHPT patients without hypertension. Active treatment for hypertension may help PHPT patients with these diseases improve their cardiac structure.

Relatively few studies have investigated RWT alterations in PHPT patients. The present study found no significant difference in RWT between the PHPT and healthy control groups, consistent with previous investigations (9, 14). Furthermore, serum phosphorus was shown to be negatively linked to RWT. The examination of RWT paired with LVMI revealed that, while the RWTs of the majority of PHPT patients were less than 0.42, a few patients might have borderline concentric remodeling (RWT greater than 0.42 with normal LVMI). It had been demonstrated that left ventricular concentric remodeling was more common in hypertensive patients. One-third of the patients in a population-based sample of subjects with moderate hypertension had left ventricular concentric remodeling; for patients with uncomplicated mild hypertension, it was an independent predictor of cardiovascular disease (25). In our investigation, we discovered higher RWT in the PHPT with hypertension group comparing to the PHPT without hypertension group. As a result of the high prevalence of hypertension in PHPT, we hypothesized that concentric remodeling in our research was connected to hypertension. Thus we recommended that clinicians paid attention to blood pressure and ventricular concentric remodeling in PHPT patients.

While we discovered lower LVEF in the PHPT groups than in the control groups, there was no significant difference in LVEF between the PHPT subgroup without hypertension and diabetes and the control groups. Furthermore, there was no correlation between laboratory parameters and LVEF. These findings are consistent with previous research. Yilmaz et al. (26) discovered that LVEF did not differ significantly between asymptomatic PHPT and control groups with matched cardiac risk factors. Agarwal (13), in a prospective case-control study, found no significant difference in LVEF between normotensive symptomatic PHPT patients and healthy controls. Satu et al. (27) discovered that PHPT patients had lower LVEF than healthy controls, although the cardiovascular disease of the former was not ruled out. The findings suggested that in PHPT patients, the deterioration of systolic cardiac function caused by other factors outweighed the positive inotropic effect of serum calcium. Another possible explanation might be the age variations across studies. The mean age of PHPT patients in Satu’s study was older than that in other studies, and aging reduced the capacity of the heart to pump blood. The third possible explanation for this discrepancy was that most studies had relatively small sample sizes. Therefore, clinical studies with large sample sizes are needed.

In addition, we found significantly lower E/A in PHPT patients compared with controls, indicating diastolic dysfunction. Diastolic dysfunction was also linked to higher serum calcium, PTH, 24-hour urine calcium, and lower serum phosphorus levels. These findings were consistent with previous studies. Purra et al. (8) discovered that patients with symptomatic PHPT had significantly lower early to late mitral annular velocity compared with matched controls for age, gender, and cardiovascular risk factors. Yılmaz et al. (26) found that E/A was significantly lower in asymptomatic PHPT than in controls after controlling for cardiovascular risk factors. Furthermore, a recent study (8) demonstrated that PHPT patients had lower E velocity and E/A than controls, and serum calcium was significantly negatively correlated with E/A. The major causes of diastolic cardiac function impairment are decreased left ventricular active diastolic performance and increased left ventricular stiffness. In PHPT patients, abnormal serum calcium and phosphorus metabolism are crucial in the pathogenesis of diastolic cardiac dysfunction. Calcium overload of cardiomyocytes impairs mitochondria and other structures of cardiomyocytes, causing necrosis, apoptosis, and subsequent fibrosis (28). PTH causes cardiac dysfunction *via* different mechanisms. PTH may boost aldosterone synthesis *via* direct or indirect mechanisms, which may have a deleterious influence on diastolic dysfunction (29). A previous study showed that the PTH receptor was expressed in the adrenal gland (30). PTH could directly activate renin-angiotensin-aldosterone by binding to PTH/PTH-related peptide receptors (31). On the other hand, PTH indirectly stimulates aldosterone secretion by increasing serum calcium levels. In other aspects, higher PTH levels exacerbate arterial stiffness, endothelial dysfunction, and arterial hypertension (32), all of which could contribute to diastolic cardiac dysfunction.

One of the strengths of this study is that we provide echocardiography data from the largest cohort of PHPT patients to date. This is the first time in mainland China that the echocardiographic characteristics of PHPT patients have been studied. However, there are some limitations to our investigation. Firstly, the study is limited by its retrospective design. The examination of changes in cardiac structure and function during long-term follow-up and the inferences of causality could not be validated. In addition, E/A cannot be solely used to determine the severity of diastolic dysfunction in patients. However, the decrease in E/A is a clinically significant indication that patients have diastolic dysfunction, particularly grade I diastolic dysfunction. An in-depth evaluation of diastolic function should be realized by comprehensive echocardiography in future studies. Furthermore, despite controlling for hypertension and other cardiovascular risk factors, not all cardiovascular diseases were excluded from PHPT. Third, we lacked particular laboratory data on calcium phosphate metabolism in controls. Additionally, because this study included only hospitalized PHPT patients with comprehensive clinical data, its generalizability might be compromised. Last but not least, there is a lack of post-parathyroidectomy echocardiographic examinations of the effect of parathyroidectomy on cardiac structure and function.

## Conclusion

Diastolic cardiac dysfunction and possible cardiac structural abnormalities, which tend to be centripetal remodeling, are discovered in this group of Chinese PHPT patients. Serum calcium, phosphorus, and PTH levels are shown to be strongly related to various cardiac structure and function parameters, lending credence to the influence of PHPT on the cardiovascular system. Our findings suggest that cardiovascular disease should be a concern among PHPT patients, and the clinical significance of cardiac dysfunction needs to be investigated further. In addition, PHPT patients should manage their blood pressure and blood glucose levels to reduce cardiac structure and function damage.

## Data availability statement

The original contributions presented in the study are included in the article/supplementary material. Further inquiries can be directed to the corresponding authors.

## Ethics statement

The studies involving human participants were reviewed and approved by The Ethics Committee of Peking Union Medical College Hospital. Written informed consent for participation was not required for this study in accordance with the national legislation and the institutional requirements.

## Author contributions

RC designed, analyzed, and wrote the manuscript, AS conducted data collection, OW reviewed, YJ, ML and WX conducted data interpretation, and XL and XX designed and reviewed the manuscript. All authors contributed to the article and approved the submitted version.
